# Thermoelectric Properties of Sb-S System Compounds from DFT Calculations

**DOI:** 10.3390/ma13214707

**Published:** 2020-10-22

**Authors:** Hailong Yang, Pascal Boulet, Marie-Christine Record

**Affiliations:** 1 Campus St Jérôme, Aix-Marseille University, CNRS, Madirel, 13013 Marseille, France; hailong.yang@etu.univ-amu.fr; 2 Campus St Jérôme, Aix-Marseille University, University of Toulon, CNRS, IM2NP, 13013 Marseille, France; m-c.record@univ-amu.fr

**Keywords:** chalcogenides, thermoelectric, DFT, QTAIM, transport properties, structure-properties relationships

## Abstract

By combining density functional theory, quantum theory of atoms in molecules and transport properties calculations, we evaluated the thermoelectric properties of Sb-S system compounds and shed light on their relationships with electronic structures. The results show that, for Sb_2_S_3_, the large density of states (DOS) variation induces a large Seebeck coefficient. Taking into account the long-range weak bonds distribution, Sb_2_S_3_ should exhibit low lattice thermal conductivity. Therefore, Sb_2_S_3_ is promising for thermoelectric applications. The insertion of Be atoms into the Sb_2_S_3_ interstitial sites demonstrates the electrical properties and Seebeck coefficient anisotropy and sheds light on the understanding of the role of quasi-one-dimensional structure in the electron transport. The large interstitial sites existing in SbS_2_ are at the origin of phonons anharmonicity which counteracts the thermal transport. The introduction of Zn and Ga atoms into these interstitial sites could result in an enhancement of all the thermoelectric properties.

## 1. Introduction

In a preceding paper [1], we performed a chemical bonding analysis on the ternary Cu-Sb-Se system compounds and showed that the weak interactions, either in local or whole structure, played an important role in lattice thermal conductivity. Since the crystal structure is related to the electronic structure and to the electronic transport path, the analysis of chemical bonds is a bridge between the structure and the thermoelectric properties.

To date, there have been many attempts that tried to explore structure and properties using the concept of chemical bonds, such as the established models of covalent bonding [2], dielectric constants [3], partially ionic binding [4,5] and ionicity [6]. Nonetheless, the complex correlation among physical interactions and bond properties makes the development of a general model very difficult. A commonly accepted concept is that the bonding information can be directly obtained from the charge density. Incontestably, the quantum theory of atoms in molecules (QTAIM) developed by Bader et al. [7,8,9,10] is the most comprehensive density-based topological tool for chemical bonding studies. Bader’s quantum theory of atoms in molecules (QTAIM) supplies a well-defined procedure of partitioning a molecule into atomic regions with zero-flux surfaces as boundaries, i.e., the surfaces on which all points satisfy,

(1) $$\nabla \rho \left(r\right)\cdot n\left(r\right)\;=0,$$

where n is a unit normal vector of the surface, and then the topology of electron density $\rho \left(r\right)$ can be characterized, by analyzing its gradient vector field.

The topological properties of a crystal charge distribution are defined by its critical points (CPs). These are points at which the gradient vector field $\nabla \rho \left(r\right)$ vanishes. In stable structures, there are four types of critical points. The CP that exists between each pair of atoms’ neighbors is called a bond critical point (BCP). The chemical bonding analysis relies on the account of the characteristics of the topological bond path between atoms, including the density $\rho \left(r\right)$ and Laplacian $\nabla^{2}\rho \left(r\right)$ at the BCP. According to these concepts, the atomic interactions can be classified into the following two different categories: the shared shell interactions characterized by $\nabla^{2}\rho \left(r\right)<0$ at the BCP and the closed shell interactions characterized by $\nabla^{2}\rho \left(r\right)>0$ at the BCP. However, the situation of bonding classification is more complex, especially in semiconductor compounds. This complex situation can be described by supplementing the information obtained from the Laplacian of the electron density with that of the following equations, the first one being derived from the local virial theorem, where G, V, and H are the kinetic, potential and total energy densities, respectively:

(2) $$\frac{\hbar^{2}}{4m}\nabla^{2}\rho \left(r\right)=2G\left(r\right)+V\left(r\right),$$

(3) $$G\left(r\right)+V\left(r\right)=H\left(r\right).$$

In the present work, our purpose is to investigate how interatomic interactions affect the thermoelectric effects in all the binary Sb-S system intermediate compounds reported in the literature by combining density functional theory (DFT), quantum theory of atoms in molecules (QTAIM), and transport properties calculations.

According to the phase diagram, the Sb_2_S_3_ stibnite is the only condensed stable binary phase in the Sb-S binary system [11,12]. This compound has been largely investigated for photovoltaic [13,14,15,16,17,18,19] and photocatalysis [20,21,22,23,24] applications, its thermoelectric properties being much less studied [25,26]. A metastibnite mineral, which has the approximate composition of Sb_2_S_3_ and contains metals in small amounts, has, however, been reported in the literature [27,28,29]. Therefore, first, we investigated the thermoelectric properties of the pure Sb_2_S_3_ crystal, and then the influence of an alloying element on these properties. The beryllium was chosen, since as a metal, it bears the smallest atomic radius. An additional compound of the Sb-S system, the tetragonal SbS_2_, was devised by calculations [30]. This compound contains interstitial sites large enough to accommodate foreign metal atoms. As with Sb_2_S_3_, we investigated both the pure SbS_2_ and the alloyed compound. In order to reveal the influence of d and p orbitals of the metal, while keeping a small atomic radius of the foreign atom, we considered zinc and gallium elements to be alloying metals.

Our investigation included topological analysis of electron density, band structures, and electronic and thermoelectric properties calculations, the latter being considered with respect to chemical potential and doping level.

## 2. Methods

### 2.1. DFT Calculations

The Perdew–Burke–Ernzerhof (PBE) [31] and generalized gradient approximation (GGA) exchange-correlation functionals were used with the Quantum Espresso (QE) package [32,33]. This package implements plane waves to develop the wave functions of the valence electrons and the projector augmented plane waves (PAW), including scalar-relativistic effects, as pseudo-potentials. The electron density for all the systems of interest was calculated with a kinetic energy cutoff of 48 Ry for the wavefunctions and 480 Ry for the electron density, and the Monkhorst–Pack procedure was used to generate *k*-points for the Brillouin zone sampling. After convergence tests, a grid of 4 × 4 × 4 *k*-points was found to be fine enough to minimize the structures parameters. The optimized structure parameters and calculated electronic gaps are summarized in Table 1. Good agreement was observed among the experimental lattice parameters of Sb_2_S_3_ (a = 11.311(1) Å, b = 3.8389(3) Å, c = 11.223(1) Å and V = 487.31(7) Å^3^) [34] and the calculated parameters. For the other compounds, the experimental lattice parameters are not available in the literature. Regarding the SbS_2_ compound, we made several structural optimizations on the starting structure extracted from the Materials Project (MP) database (a = b = 6.061 Å, c = 11.59 Å, and V = 426.02 Å^3^) [30] and found several distinct structures with different lattice constants. We chose to keep the most stable structure (i.e., with the lowest energy, which was about 0.3 eV lower than that from MP) for the subsequent calculations. The Sb_2_S_3_ and SbS_2_ optimized structures were used as the starting point in the optimization process of the alloyed structures.

### 2.2. Electronic Transport Calculations

Once the electron density was obtained for the optimized structure of interest, the *k*-point grid was tremendously enlarged to calculate the thermoelectric transport properties. As a rule of thumb, the density was calculated on a grid comprising several thousands (six to ten thousand) *k*-points. The transport properties were calculated with the BoltzTraP program [35].

### 2.3. QTAIM Calculations

The electron density was analyzed with the program Critic2 [36] that implemented Bader’s quantum theory of atoms in molecules (QTAIM). In this work, the model used in Critic2 for the kinetic energy density $G\left(r\right)$ calculation was based on the Thomas–Fermi equation with the semiclassical gradient correction proposed by Kirzhnits [37,38], and further refined by the approximate Abramov [39] expression for directly relating $G\left(r\right)$ to $\rho \left(r\right)$:

(4) $$G\left(r\right)=\frac{3}{10}{\left(3\pi^{2}\right)}^{\frac{2}{3}}\rho {\left(r\right)}^{\frac{5}{3}}+\frac{1}{72}\frac{{\left[\nabla \rho \left(r\right)\right]}^{2}}{\rho \left(r\right)}+\frac{1}{6}\nabla^{2}\rho \left(r\right).$$

In turn, the potential energy density was determined using the local Virial equation from the knowledge of $G\left(r\right)$ and $\nabla^{2}\rho \left(r\right)$.

On the basis of the properties of Laplacian distribution and total energy density, the local properties at the BCP could be used to analyze a series of interactions in solids or crystals between pure closed-shell interactions and shared-shell interactions. According to Equation (3),

(5) $$\frac{H_{b}}{\rho_{b}}=\frac{G_{b}}{\rho_{b}}\left(1-\frac{\left|V_{b}\right|}{G_{b}}\right).$$

When ∇ρ vanishes at the BCP, $G_{b}$, $V_{b}$, and $H_{b}$ are only related to $\rho_{b}$ and $\nabla^{2}\rho \left(r\right)$ from Equations (4) and (2). Equation (5) displays the variation of $H\left(r\right)/\rho \left(r\right)$ along with $\left|V_{b}\right|/G_{b}$. The former indicator, the bond degree first introduced by Espinosa [40], stands for the total energy per electron related to the total electronic pressure, and the latter indicator exhibits the competition between potential energy and kinetic energy for bonding formation. These two indicators, which correspond to specific characteristics of bonding, can be used for the bonding classification. At the same time, the kinetic energy per electron $G\left(r\right)/\rho \left(r\right)$ is related to the variation tendency of $H_{b}/\rho_{b}$ vs. $\left|V_{b}\right|/G_{b}$, which is also an important parameter for classification of bonding [40,41,42].

The crystal structures have been visualized with VESTA [43].

## 3. Results and Discussion

### 3.1. The Sb_2_S_3_ Compound

#### 3.1.1. Pure Sb_2_S_3_

**Figure 1:**
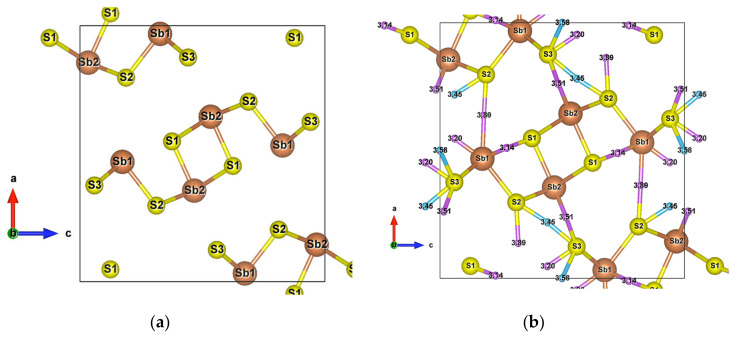
(**a**) Unit cell of Sb_2_S_3_ (Pnma); (**b**) Crystal structure of Sb_2_S_3_ showing interactions with the second nearest atoms (Sb–S and S–S). Purple and sky-blue small balls are the bond critical points (BCPs) for Sb–S interactions and S–S interactions, respectively.

Due to its crystal structure made of one-dimensional (1D) ribbons of polymerized [Sb_4_S_6_]_n_, the low-dimensional Sb_2_S_3_ is of wide interest in various applications, such as in television cameras [44], microwave devices [45], switching devices, [46] various optoelectronic devices, [47,48,49], as well as photoelectric and thermoelectric cooling devices [50,51,52]. This compound crystallizes into an orthorhombic structure (Pnma) [34], which has two and three different atomic positions for Sb and S, respectively. The Sb1 atoms and Sb2 atoms are located at the centre of a pyramid made by the closest S atoms, a trigonal pyramid for Sb1 and a tetragonal pyramid for Sb2, which together form the infinite [Sb_4_S_6_]_n_ ribbons parallel to the *y*-axis (Figure 1a).

**Figure 2:**
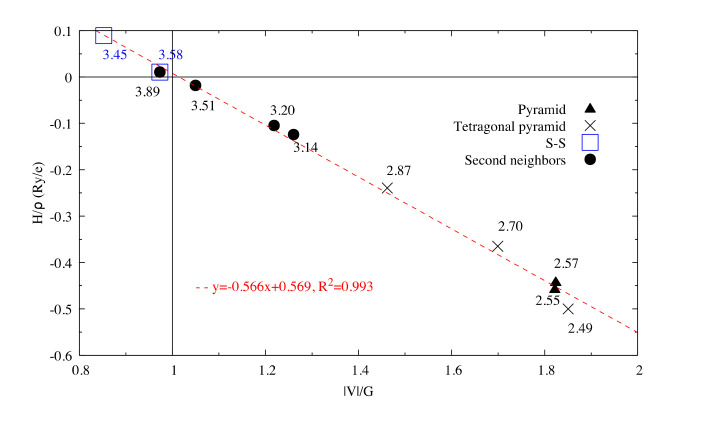
$H_{b}/\rho_{b}\;\mathrm{vs}.\;\left|V_{b}\right|G_{b}$ for interactions in Sb_2_S_3_. The dash line corresponds to the fitting line for Sb–S interactions by least square method. The numbers related to the different points correspond to the bond length (in Å).

The [Sb_4_S_6_]_n_ chains are linked by longer Sb–S interactions and S–S interactions (Figure 1b), forming the whole stable crystal structure. Taking into account the closest and second closest atoms, the coordination numbers are (3 + 4)-fold, (5 + 2)-fold, (3 + 2)-fold, (3 + 2)-fold, and (2 + 6)-fold for Sb1, Sb2, S1, S2, and S3 atoms, respectively. Among the interatomic interactions with the second closest atoms (Figure 2), the Sb1–S1 (3.14 Å), Sb1–S3 (3.20 Å), and S3–S3 (3.58 Å) bonds connect the adjacent ribbons along the *z*-axis, while the remaining interactions link ribbons in the *x* direction. Due to these distributions of interatomic interactions, the electronic transport is obviously anisotropic. The electronic motion is preponderant in the *y* direction and worst in the *x* one (Figure S1) but the doping type has a different impact on the electrical conductivity ratio depending on the direction.

For Sb_2_S_3_, the experimental energy gap has been reported to lie between 1.61 to 3.8 eV [13,34,50,51,52,53,54]. The calculated band gaps for bulk Sb_2_S_3_ are underestimated relative to these measured values. The value of 1.3 eV, calculated in this work, is in good agreement with the values of 1.29 eV and 1.35 eV determined by GGA-PAW and GGA calculations, respectively [55,56], whereas the local-density approximation (LDA) calculation led to a larger band gap of 1.55 eV [57].

**Figure 3:**
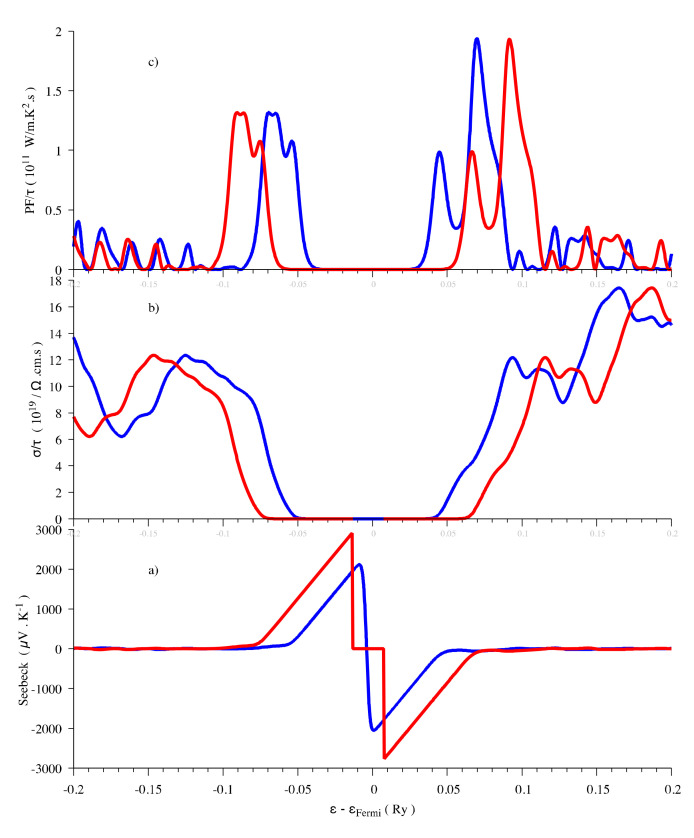
Thermoelectric properties (Seebeck coefficient (**a**), electrical conductivity (**b**) and power factor (**c**)) as a function of the chemical potential at 300 K for Sb_2_S_3_. Red curve, Eg = 1.88 eV and blue curve, Eg = 1.3 eV.

The Engel–Vosko functional and Tran-Blaha modified Becke-Johnson (TB-mBJ) potential can both result in an enlarged band gap that is 1.76 eV for the former and 1.88 eV for the latter [26,58]. In any case, the GGA and LDA calculations are expected to underestimate the gaps [59]. In order to investigate the influence of the band gap on the thermoelectric properties, we performed the thermoelectric properties calculations by using both 1.3 eV and 1.88 eV as band gap energies, the latter one being obtained with the help of a scissor operator. The shapes of the evolution are similar, however, the maximum values of the Seebeck coefficient are different (Figure 3). In agreement with the results calculated with the TB-mBJ approximation [26], the Seebeck coefficient has maximum values of around ±3000 μV/K near the Fermi level at 300 K when the gap is 1.88 eV, even if, in the present work, we used a denser mesh in the Brillouin zone (3000 vs. 2000 *k*-points). It can be concluded that the band gap does not influence the thermoelectric properties evolution vs. chemical potential. Only the maximum values of Seebeck coefficient calculated from Boltzmann theory are affected. The underestimation of the band gap results in an underestimation of the Seebeck coefficient that can be corrected by applying a scissor operator.

**Figure 4:**
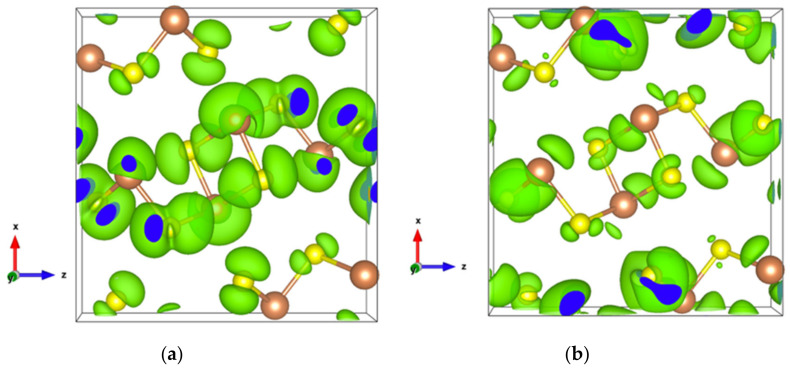
Electronic density of Sb_2_S_3_ with an isosurface of 0.0005 electrons/bohr^3^. (**a**) For the highest occupied orbital; (**b**) For the lowest unoccupied orbital.

The very high Seebeck coefficient of Sb_2_S_3_ originates from the very abrupt density of states (DOS) of valence bands, which are dominated by the Sb-5p and S-3p orbitals [26,55,56,57,58]. Even if the Sb–S and S–S interactions involving second nearest atoms are weak (see Figure 2), they largely contribute to the DOS near the Fermi level. In effect, the antibonding state of long bonds tends to be filled by electrons and the long range order takes advantage of the largest contributions of S-3p, and also induces astereochemical activity of Sb-5s lone-pair electrons at the top of the valence band. This can be seen from the electronic density of the highest occupied band in Figure 4a. By comparing with Figure 1, we see that S2 and S3 atoms have larger contributions to the electronic density than S1 atoms that have the shortest second nearest neighbor Sb–S bonding, located within the ribbons. The lone-pair electrons on Sb1 atoms have more room than those on Sb2 atoms, which have shorter Sb–S interactions; this indicates that the lone-pair electrons of Sb2 atoms are more stereochemically inert. Moreover, these weaker interactions with delocalized electron state surrounding the ribbons result in the confinement of the dominant electron state within the ribbon and display the quasi-one-dimensional structure of [Sb_4_S_6_] (Figure 4). In addition, the soft bonds located close to the $\left(1,0\right)$ point in Figure 2 involve almost all the atoms in the cell, implying a range of low frequency vibrations in the crystal, thus, resulting in low lattice thermal conductivity.

At the same time, n-type doping and p-type doping both display a good thermoelectric performance in the whole temperature range (Figure S2a), both reaching the optimized thermoelectric power factor around the carrier concentration of ±1 × 10^21^ e/cm^3^. However, the n-type thermoelectric performance is severely degraded with further increase in the doping concentration, while the PF of the p-type compound is gradually reduced. The reason is that the Seebeck coefficient has a prominent reduction with the increase in carrier concentration for the n-type compound, whereas the electrical conductivity is substantially enhanced with the increase in carrier concentration for both n- and p-type compounds (Figure S2b). Irrespective of the carrier concentration up to ±1 × 10^21^ cm^−3^, it is noticeable that the n-type doping has a larger advantage over the p-type doping. Moreover, the PF exceeds 1 at 300 K and reaches 6.6 at 800 K when doping with 1 × 10^21^ e/cm^3^. Taking into account that this compound should have a relatively low thermal conductivity due to soft bonds in the whole cell (see above), Sb_2_S_3_ is promising for thermoelectric applications.

**Figure 5:**
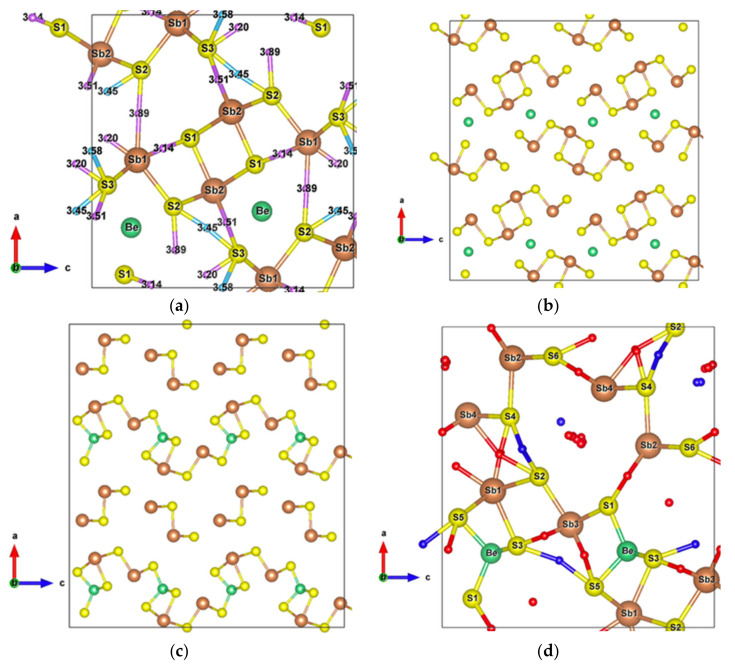
(**a**) Sb_2_S_3_ unit cell with inserted Be metal atoms along the *z*-axis; (**b**) Sb_2_S_3_ supercell with Be atoms; (**c**) Sb_2_S_3_ supercell with Be atoms after structural relaxation; (**d**) Relaxed Sb_2_S_3_ unit cell with all interatomic interactions. Small red balls are BCPs of Sb–S interactions and blue balls are BCPs of S–S secondary neighbors interactions.

Having plotted all the bonds in the cell (Figure 1b), an interstitial site between the ribbons surrounded by a six-membered ring -Sb1-S2-Sb1-S1-Sb2-S3- clearly appears. The packing ratio of the cell is about 29% relative to atomic radii, indicating enough space for introducing impurities. Moreover, the existence of the metastibnite mineral in the Sb-S system, which has the approximate composition of Sb_2_S_3_ and contains metals in small amounts [27,28,29], allows one to envisage the adding of impurities into the Sb_2_S_3_ crystal. The [Sb_4_S_6_]_n_ chains, which are parallel to the *y*-axis, result in a higher electrical conductivity in this direction (Figure S1). The insertion of metal atoms, in the interstitial sites described above, generates a new chain of atoms along the *z*-axis that creates infinite [Sb_4_S_6_M_2_]_n_ chains along this axis by connecting adjacent [Sb_4_S_6_] ribbons (Figure 5a,b). In order to keep the original [Sb_4_S_6_]_n_ chains and the semiconducting properties of the material, the light and small Be atom is chosen to be inserted into the cell interstitial sites.

#### 3.1.2. The Be-Sb_2_S_3_ Alloy

**Figure 6:**
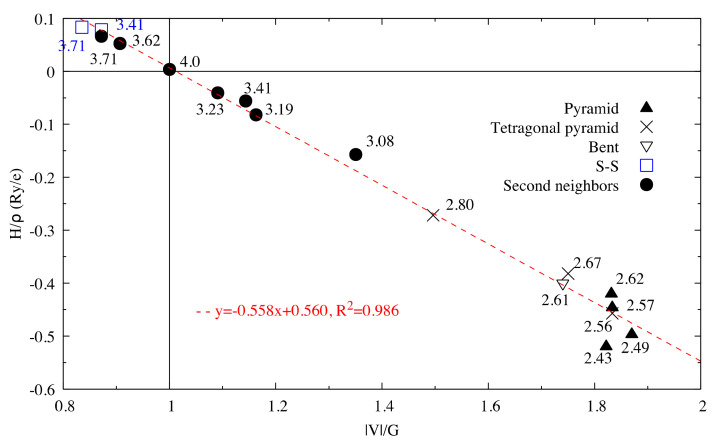
$H_{b}/\rho_{b}$ vs. $\left|V_{b}\right|/G_{b}$ for interactions in optimized Sb_2_S_3_Be_2_. The dash line corresponds to the fitting line for Sb–S interactions by least square method.

After structural relaxation, the original Sb_2_S_3_ structure is modified, resulting in the formation of pyramidal Be, which connects adjacent [Sb_2_S_7_] groups and leaves the [Sb_2_S_4_] groups away from the original [Sb_4_S_6_] ribbons, as shown in Figure 5c. The alternate layered structures along the *x*-axis consist of [Sb_2_S_4_]_n_ chains parallel to the *y*-axis and infinite [Sb_2_S_9_Be]_n_ chains along the *z*-axis. According to the topological analysis of chemical bonding (Figure 6), among all the interatomic interactions displayed in Figure 5d, there are four and six different atomic environments for Sb and S atoms, respectively. The Sb1 atoms belong to a square pyramid, the Sb3 and Be atoms belong to a triangular pyramid, and all these polyhedra are contained in the [Sb_2_S_9_Be] group. The [Sb_2_S_4_] group includes a triangular pyramid containing Sb2 atoms, and Sb4 atoms with two out of the three coordinations, which can be called “bent” coordinations with almost a right angle. In this new cell, in addition to the weaker interactions between secondary neighbors plotted in Figure 6, there are Sb–S and S–S long range interactions (about 5 Å), the BCPs of which are surrounded by the Sb-S nine-membered ring shown in Figure 5d.

**Figure 7:**
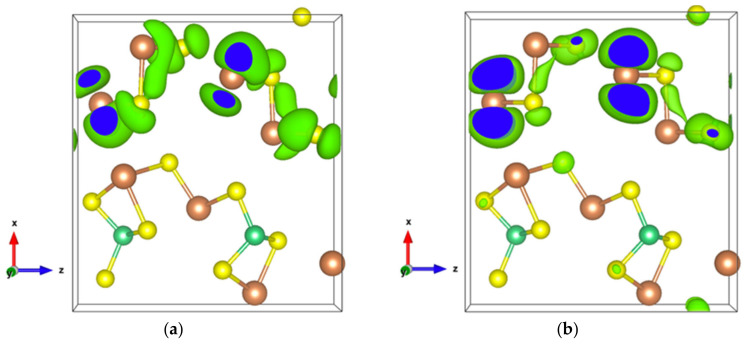
(**a**) Electronic density of Sb_2_S_3_Be_2_ for the highest occupied orbital with an isosurface of 0.001 electrons/bohr^3^; (**b**) Electronic density of Sb_2_S_3_Be_2_ in the energy range comprising the top two valence orbitals with an isosurface of 0.003 electrons/bohr^3^.

The energy band structure, given in Figure S3, shows that the two orbitals localized at the top of the valence band almost separate from the other valence orbitals. In addition, the electron density of states of the highest occupied orbital (Figure 7a), as well as that of the top two valence orbitals (Figure 7b), are localized in the [Sb2S4]_n_ chains. As can be seen in Figure 7b, the Sb4-p and S6-p orbitals dominate the electron states of the top two valence orbitals, and the electron density of both Sb4-p and S6-p, which forms a kind of dumbbell, extends along directions so as to form weaker bonds, for example, the Sb4-S6 bonds with a bond length of 3.19 Å, which link the [Sb2S4]_n_ chains along the *z*-axis. Nevertheless, the electron transport path along the *x*-axis must pass through the triangular pyramid made of Be, S1, S3, and S5 atoms, which has very poor electron contributions to the DOS near the Fermi level, especially the Be atoms with almost empty states around the Fermi level (Figure S4). The localized electron states in [Sb2S4]_n_ chains and the electron-poor triangular pyramid both severely restrict the electron transport along the *x*-axis, resulting in an almost null electrical conductivity in the *x* direction with p-type doping (Figure S5a). Since the electron density in the top of the valence band along the *y-* and *z*-axes is equivalent, without a region with low density, the electrical conductivity is the same in these two directions for p-type doping (Figure S5a). For n-type doping, the conductivities along *y-* and *z*-axes are different.

Obviously, the interatomic bonding along the *y*-axis is stronger than that along the other two axes, where weak bonds between [Sb2S4]_n_ chains and [Sb2S9Be]_n_ chains are located. This is why the electrical conductivity along the *y*-axis is much higher than that along the other two axes, being similar for electron doping at the bottom of the conduction band.

The Seebeck anisotropy is more complex, and more sensitive to carrier transport and temperature than that of the electrical conductivity; in addition, the scattering mechanisms play an important role [60].

Along the *x*-axis, the carriers proceed on a path containing weak bonds between layers and pyramidal Be, whereas along the *y*-axis only the [Sb2S9Be]_n_ chains that stand the pyramidal Be are concerned. Along the *z*-axis, there are different competing paths, the [Sb2S4]_n_ chains with weak bonds and the [Sb2S9Be]_n_ chains containing pyramidal Be, which should result in different scattering mechanisms for specific carriers and temperature. The Seebeck effect is more prominent along the *z*-axis and the *x*-axis for p-type doping and n-type doping, respectively, under the conditions of low carrier concentration and room temperature, as shown in Figure S5b.

Although Sb_2_S_3_Be_2_ exhibits an anisotropy in the electronic and thermoelectric properties, the total Seebeck coefficient and the thermoelectric power factor are not as high as those of Sb_2_S_3_ (Figure S6). The main reason is that the electron density at valence band top is confined in the [Sb_2_S_4_]_n_ chains, making smaller contributions to DOS at the Fermi level (Figure S7). The extrema of the Seebeck coefficient are −653 μV/K and 595 μV/K at 300 K for the chemical potential of +0.06 eV and −0.065 eV relative to the Fermi level, respectively. These values could be underestimated due to the underestimation of the band gap by GGA calculations. It can be assumed from these extrema values that the n-type doping is more effective than the p-type doping for thermoelectric applications. Calculations of the power factor and the Seebeck coefficient for various carrier concentrations ranging from 5 × 10^18^ to 5 × 10^21^ cm^−3^ for both p- and n-type doping (Figure S8) show, irrespective of the carrier concentration, better properties for electron doping. For carrier concentrations less than or equal to 1 × 10^20^ cm^−3^, the maximum value of these properties, at a temperature that increases with the carrier concentration, is evidenced. The highest thermoelectric power factor is obtained for the carrier concentration of 1 × 10^21^ e/cm^3^, irrespective of the temperature, and its substantial decrease in higher concentrations results from the strong decrease in the Seebeck coefficient. The power factor obtained for 5 × 10^20^ e/cm^3^ is very close to that for 1 × 10^21^ e/cm^3^, for temperatures above 700 K. At lower temperatures, the power factor for 5 × 10^20^ e/cm^3^ is lower than that for 1 × 10^21^ e/cm^3^, this difference being related to higher Seebeck coefficient values below 400 K for 1 × 10^21^ e/cm^3^, and especially along the *x* direction for temperatures below 500 K (Figure S9).

As for p-type doping, the power factor is lower than that for n-type doping, its highest values being obtained for a carrier concentration of 5 × 10^20^ holes/cm^3^ in the whole temperature range. The decrease in the power factor for highest carrier concentrations is related to the decrease in the Seebeck coefficient. Moreover, the anisotropy of the Seebeck coefficient for p-type doping is less pronounced than that for n-type doping.

### 3.2. The SbS_2_ Compound

#### 3.2.1. Pure SbS_2_

**Figure 8:**
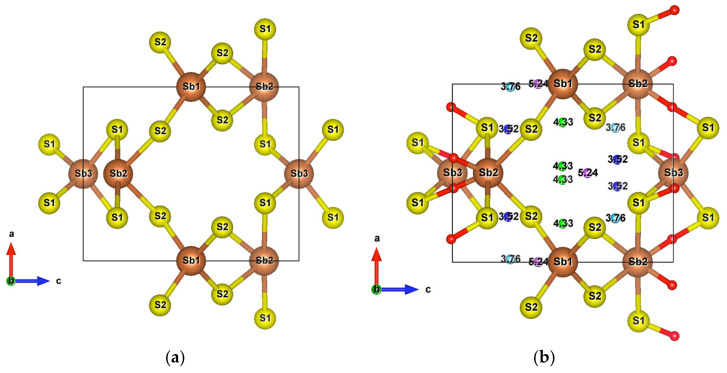
(**a**) Unit cell of SbS_2_; (**b**) Crystal structure of SbS_2_ showing weak interactions. The red small balls are BCPs of Sb–S interactions and the remaining with other colors are BCPs of S–S interactions.

**Figure 9:**
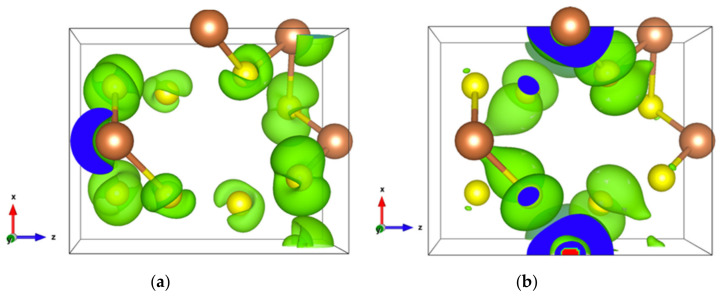
Electronic density of SbS_2_. (**a**) In the energy range comprising the top two valence orbitals with an isosurface of 0.004 electrons/bohr^3^; (**b**) For the single bottom conduction orbital with an isosurface of 0.003 electrons/bohr^3^.

Infinite SbS_2_ strings constituted of ψ-SbS_3_ tetrahedrons and ψ-SbS_4_ bipyramids have been found in BaSb_2_S_4_ [61]. SbS_2_ has also been synthesized under glass, with a glass transition temperature at 163 °C, and nanocrystalline thin films (Mo:SbS_2_) forms [62,63]. The crystal structure of SbS_2_ has been devised by calculations [30]. SbS_2_ has a tetragonal structure and crystallizes in the $P\bar{4}$ group. It contains four formula units in the conventional cell for which the optimized lattice parameters are a = 6.56 Å and c = 8.14 Å. This structure exhibits three different positions for the Sb atoms and two for the S atoms, these atoms constituting different [SbS_4_] polyhedrons. The [Sb2S1_2_S2_2_] bipyramids are linked to two [Sb1S2_4_] and two [Sb3S1_4_] tetrahedrons, and both [Sb1S2_4_] and [Sb3S1_4_] tetrahedrons are surrounded by four [Sb2S_4_] bipyramids, which constitute the basic crystal framework of six-membered rings, i.e., six polyhedrons of [Sb2S_4_]-[Sb1S_4_]-[Sb2S_4_]-[Sb3S_4_]-[Sb2S_4_]-[Sb1S_4_], extending to the whole space to form large interstitial sites at 1a and 1d positions, as shown in Figure 8a. A topological analysis of chemical bonds shows that the Sb2 atoms form with S1 atoms, two weak bonds with a bond length of 3.26 Å. The S atoms interact with each other across the interstitial sites, the S2–S2 interactions having longer interatomic distances of 4.33 Å and 5.24 Å. than those of S2–S1 interactions that are equal to 3.52 Å and 3.76 Å (Figure 8b). The electronic band structure (Figure S10a) calculated with the GGA approach shows that the lowest unoccupied band locates at the A point, whereas the highest occupied band is between the Z and R points, indicating that SbS_2_ is an indirect semiconductor with a band gap of 0.66 eV. The relatively low dispersive two top valence orbitals interact only with the other valence orbitals around the G point and are mainly contributed by the Sb2-s and S1-p orbitals and to a lesser extent by the S2-p orbital. The electron density of S1-p and S2-p extends towards the Sb2 atoms and that of Sb2-s towards the S1 atoms (Figure 9a), corresponding to the Sb-S bonds in [Sb2S_4_] bipyramids and the bond between second nearest atoms Sb2-S1, respectively. The electron states of the two top valence orbitals mainly come from the [Sb2S_4_] bipyramids, especially from the nearly linear S1-Sb2-S1 fragment bearing an angle of 178.4°.

The bottom conduction orbital completely separates from the other conduction orbitals (Figure S10a) resulting in a single peak of the DOS above the Fermi level (Figure S10b). The pertaining electron states belong to the [Sb1S_4_] tetrahedron and the spindle-like electron density of S2-p extends towards Sb2 atoms (Figure 9b), indicating a contribution of Sb2-p. Thus, SbS_2_ exhibits a special electron transport path from [Sb2S_4_] bipyramids to [Sb1S_4_] tetrahedrons.

The Seebeck coefficient of SbS_2_ reaches 1150 and 1025 μV/K at the chemical potential of −0.03 and 0.07 eV relative to the Fermi level at 300 K, respectively (Figure S11a), indicating that a p-type doping is more efficient for TE applications. These results are in agreement with a larger DOS derivative observed near the Fermi level in the valence band, which is contributed by the [Sb2S_4_] bipyramids. The single peak of the electrical conductivity observed at positive chemical potential near the Fermi level and related to the single peak observed in the conduction part of the DOS, which originates from the [Sb1S_4_] tetrahedron, weakens the thermoelectric effect. As a consequence, the thermoelectric power factor is poorer for n-type doping than for p-type doping. Even at 300 K in the chemical potential range [−0.03 Ry, 0.04 Ry], which is larger than the usual doping range of ~10 k_B_∙T, the conductivity is the same along the *x*, *y*, and *z* directions (Figure S11b), an anisotropy is observed for the electrical conductivity in the remaining chemical potential ranges, in concordance with the crystal structure. This anisotropy can be explained by the electron transport path that is different along the *x*, *y* and *z* directions. The electrons must go through the six-membered polyhedral rings along the *z*-axis, whereas they have two optional paths along the *x*- and the *y*-axes. Indeed, the electrons can go through the [Sb1S_4_] tetrahedron and the [Sb2S_4_] bipyramid or the [Sb3S_4_] tetrahedron and the [Sb2S_4_] bipyramid. Obviously, the more efficient electron path in the cell is that passing through the [Sb1S_4_] tetrahedron and the [Sb2S_4_] bipyramid, corresponding to better electrical properties along the *x*- and the *y*-axes than along the *z*-axis.

To be more accurate, the thermoelectric properties investigation has to be completed by considering the influence of the doping. Although the Seebeck coefficient has similar evolution vs. temperature irrespective of the doping type, the p-type doping has a larger Seebeck coefficient than that of the n-type doping for the same carrier concentration (Figure S12a).

The Seebeck coefficient decreases with the increase in carrier concentration and behaves distinctively with respect to temperature depending on the carrier concentration. For low carrier concentrations, it increases up to around 500 K, and then drastically decreases above this temperature, whereas, for high carrier concentrations, it slightly increases with temperature to reach a plateau at high temperatures. Regarding the thermoelectric power factor (Figure S12b), the influence of the temperature differs from n-type to p-type doping.

The p-type doping yields a higher thermoelectric power factor at low temperatures, whereas the n-type doping performs better at high temperatures, the crossing point being displaced towards higher temperatures as the carrier concentration increases. For the hole concentration of 1 × 10^20^ cm^−3^, the power factor reaches its maximum at about 400 K, whereas for the same electron concentration, the power factor reaches its maximum at about 850 K. For higher carrier concentrations, the p-type doping shows better efficiency than the n-type doping in the whole temperature range of interest (0–1000 K). For both p-type and n-type doping, the maximum power factor is obtained for a carrier concentration of 5 × 10^20^ cm^−3^. Although the thermoelectric performance of SbS_2_ seems to be not as good as that of Sb_2_S_3_, the large interstitial site in the cell and the long-range S–S interactions should be at the origin of phonon anharmonicity and should result in low lattice thermal conductivity.

The packing ratio of the SbS_2_ cell is about 27% relative to atomic radii, indicating that interstitial sites at 1a and 1d Wyckoff positions are large enough to accommodate foreign metal atoms.

Two situations can occur when introducing metal atoms in these interstitial sites. The first situation occurs when the metal atoms form strong bonds with adjacent S atoms inducing a decrease in the cell volume. The second situation occurs when the metal atoms form weak interactions with adjacent S atoms and result in an increase in cell volume. The latter situation is similar to a cage structure, making the metal atoms vibrate in the cage, which greatly enhances the vibrations anharmonicity, and thus reduces the lattice thermal conductivity. Both situations are discussed hereafter.

#### 3.2.2. The Zn-SbS_2_ Alloy

The first situation can be achieved by introducing two Zn atoms at 1a and 1d positions. After structure optimization, the cell volume of SbS_2_Zn_2_ decreases by about 6.7%, resulting from an increase in a and a decrease in c parameters.

**Figure 10:**
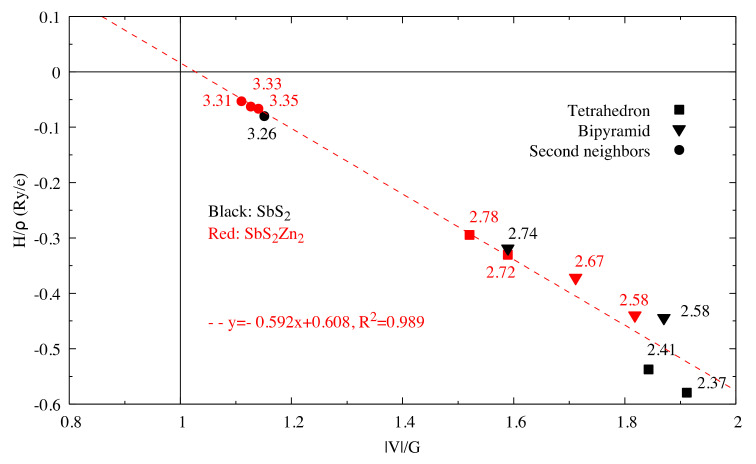
$H_{b}/\rho_{b}$ vs. $\left|V_{b}\right|/G_{b}$ for Sb–S interactions in SbS_2_ and SbS_2_Zn_2_.

The energy densities of all the Sb-S bonds in SbS_2_ and SbS_2_Zn_2_ are plotted in Figure 10.

The bond lengths in the [Sb1S_4_] and [Sb3S_4_] tetrahedrons increase as the coordinated atoms of Sb1 and Sb3 change from S2 to S1 and from S1 to S2, respectively, when the composition goes from SbS_2_ to SbS_2_Zn_2_. At the same time, the bond angle defined by S1-Sb2-S1 decreases from 178.4° to 154° and the corresponding bond lengths decrease.

**Figure 11:**
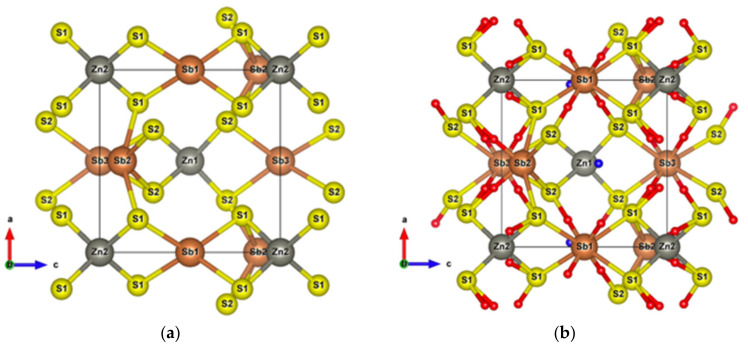
(**a**) Unit cell of SbS_2_Zn_2_; (**b**) Crystal structure of SbS_2_Zn_2_ showing all the weak interactions. Small red balls are BCPs of Sb–S interactions and the blue balls are BCPs of S–S interactions.

By contrast, the bond angle defined by S2-Sb2-S2 increases while the corresponding Sb2-S2 bond lengths remain the same, making the [Sb2S_4_] bipyramids more planar, as shown in Figure 11a. In spite of the formation of strong Zn-S bonds in the [ZnS_4_] tetrahedrons with a bond length of 2.36 Å, which is slightly longer than the sum of the atomic radii (2.3 Å), the long-range S1–S2 interactions still exist, although with elongated interactomic distances (5.69 Å), as shown in Figure 11b.

**Figure 12:**
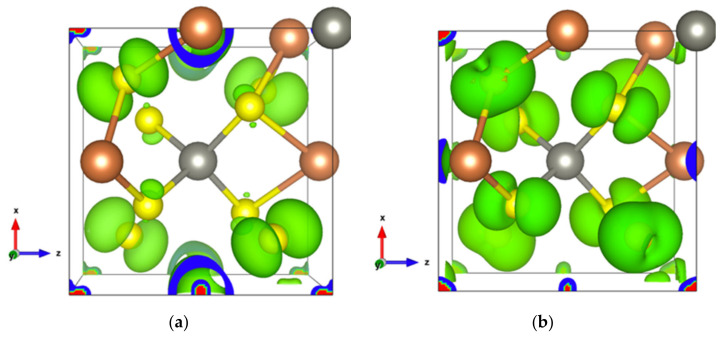
Electron density of SbS_2_Zn_2_ (**a**) In the highest occupied orbital; (**b**) In the energy range comprising the top two valence orbitals with an isosurface of 0.002 electrons/bohr^3^.

With two Zn atoms introduced at 1a and 1d Wyckoff positions, under GGA calculations, the band gap decreases from 0.66 to 0.52 eV. Some very localized bands appear in the energy range of −6 to −7 eV relative to the Fermi level (Figure S13a). These bands originate from the Zn-3d orbitals and generate a very intense DOS (Figure S13b and inset), indicating that Zn-S bonds are strong and tend to lower energy. The maximum of the highest occupied orbital, which is dominated by Sb1-s and S1-p (Figure 12a), locates at the G point. The two top valence orbitals almost separate from other bands along the high symmetry k-points’ path (Figure S13a), the electron states of which are dominated by the S1-p and S2-p orbitals (Figure 12b). The hole conduction properties are governed by these two orbitals (Figure S14a). Irrespective of the doping type, the electronic transport properties along the *x*- and *y*-axes are the same, whereas they differ from those along the *z* direction, except in the chemical potential range 0–0.68 eV. Along the *z*-axis, the optimal conduction path goes through the [ZnS1_4_] and [Sb1S1_4_] polyhedra, whereas along the *x*- and *y*-axes, it goes through a path alternating S1 and S2 atoms. Thus, the highest electrical conductivity observed along the *z*-axis for hole doping can be attributed to the dominance of S2 atoms at the top of the valence band.

Regarding the thermoelectric effect, the very low density of states at the valence band edge (Figure S13b) results in a Seebeck coefficient for hole doping lower than that for electron doping (Figure S14b). Nonetheless, the Seebeck coefficient reaches 797 and 920 μV/K at the chemical potential of −0.06 and 0.04 eV, respectively. The higher Seebeck coefficient for n-type doping is in agreement with the thermoelectric power factor calculated for various doping levels (Figure S15a). The maximum thermoelectric power factor for hole doping with the optimal carrier concentration of 1 × 10^21^ h/cm^3^ is even much lower than that with electron doping with a carrier concentration of 1 × 10^20^ e/cm^3^. However, the Seebeck coefficient for electron doping, at a low carrier concentration, drastically falls at high temperatures, as seen in Figure S15b. Overall, SbS_2_ has a good thermoelectric power factor for electron doping when introducing Zn atoms in the interstitial sites but the anharmonicity sources present in SbS_2_, namely the long range S1–S2 interactions and the secondary Sb–S interactions, are weakened.

#### 3.2.3. The Ga-SbS_2_ Alloy

**Figure 13:**
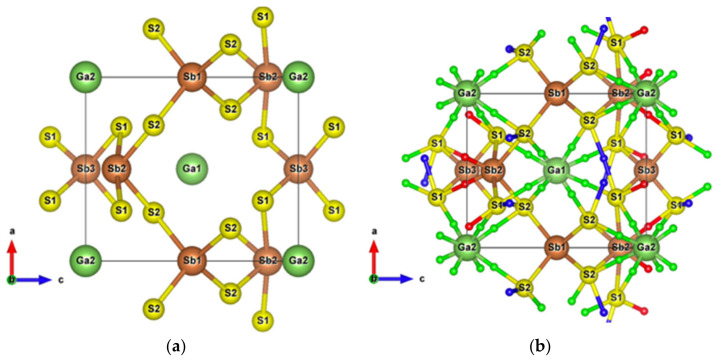
(**a**) Unit cell of SbS_2_Ga_2_; (**b**) Crystal structure of SbS_2_Ga_2_ showing the weak interactions. Small red, blue, and green balls are BCPs of Sb–S, S–S, and Ga–S interactions, respectively.

**Figure 14:**
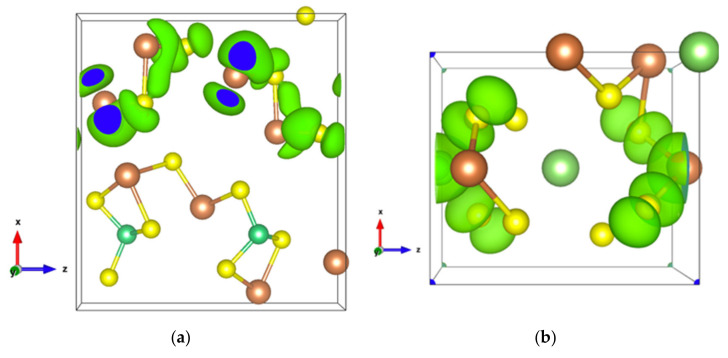
Electronic density of SbS_2_Ga_2_ with an isosurface of 0.002 electrons/bohr^3^. (**a**) For the top valence orbital; (**b**) For the bottom conduction orbital.

**Figure 15:**
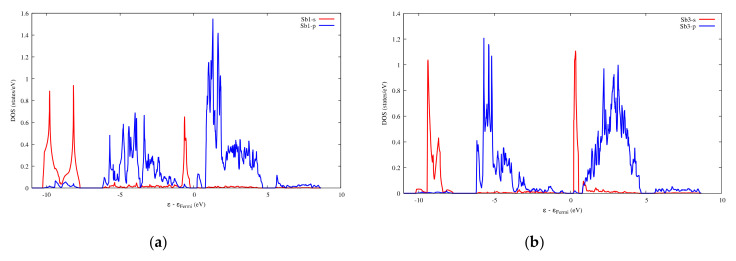
Projected DOS of SbS_2_Ga_2_. (**a**) On the Sb1 atoms; (**b**) On the Sb3 atoms.

When two Ga atoms are introduced into the 1a and 1d interstitial sites of SbS_2_, the cage structure after optimization is maintained. The volume of the new cell is increased by about 10% as the a lattice parameter and the c lattice parameter increase and decrease, respectively. The Ga atoms are surrounded by eight adjacent S atoms forming weak Ga–S interactions with interatomic distances from 3.14 to 3.38 Å (Figure 13), which are enclosed in the range delineated by the atomic radii sum (2.24 Å) and the Van der Waals radii sum (3.67 Å). Meanwhile, the interatomic distance for the weak S1–S2 interactions still reaches 3.6 Å, which is close to the sum of the Van der Waals radii. The architecture around the Sb atoms has undergone only slight changes, except that the bond length of Sb1-S2 increases by about 0.22 Å. In the band structure, the top valence orbital separates from other valence orbitals and, as observed for SbS_2_, the bottom conduction orbital is completely independent from other conduction orbitals (Figure S16). Figure 14 and Figure 15 show that the electron states of the top valence orbital and the bottom conduction orbital are dominated by Sb1-s, S2-p and Sb3-s, S1-p, respectively. These electron states belong to the [Sb1S_4_] and [Sb3S_4_] tetrahedrons, respectively, which constitute the specific electron transport path from the valence band to the conduction band.

The Seebeck coefficient for n-type doping is larger than that for p-type doping, as shown in Figure S17a. It reaches 525 and −853 μV/K at 300 K, for the chemical potential of −0.1 and 0.001 eV, respectively. Regarding the thermoelectric power factor (Figure S17b), for similar carrier concentrations up to 10^20^ cm^−3^, at low temperatures (<500 K), the PF is higher for n-type doping. For higher temperatures, the PF is higher for p-type doping irrespective of the concentrations. For both p-type and n-type doping, the PF increases with the carrier concentration, up to 5 × 10^20^ cm^−3^ and decreases beyond this value. As with SbS_2_, along the *z*-axis, the electrons must go through the six-membered polyhedral rings, whereas along both the *x*- and *y*-axes they have two possible paths depending on the doping type, namely through the [Sb1S_4_] tetrahedrons and [Sb2S_4_] bipyramids in the valence band or through the [Sb3S_4_] tetrahedrons and the [Sb2S_4_] bipyramids in the conduction band. Thus, the electrical properties along the *x*- and *y*-axes are similar and better than along the *z*-axis (Figure S18a). The anisotropy between the *x*/*y* and *z* directions is less pronounced for the Seebeck coefficient, except for the highest energy levels in the valence band (Figure S18b).

## 4. Concluding Summary

By combining the analysis of the distribution of chemical bonds through the QTAIM approach, with that of the electronic band structure, density of states, and electron density maps, a detailed description of the structure-properties relationships can be drawn. The electronic transport paths going through specific atom groups have been distinctly identified in the cell, being responsible for the isotropic or anisotropic character of the electrical properties. As for the Seebeck coefficient, the isotropy or anisotropy are much more difficult to explain, as the Seebeck is comprehensively influenced by carriers, temperature, scattering mechanisms, and lattice, which need to be discussed with specific doping.

In Sb_2_S_3_, the electronic transport is anisotropic. The Sb electron lone pairs do not have the same activity, being more or less stereochemically active, depending on their environment. Together with the fact that soft bonds involve almost all the atoms in the cell, the lattice thermal conductivity should be low.

In Sb_2_S_3_Be_2_, the structure and density of states analysis shows that, the electron transport in the *x* direction has to pass through the Be atom arranged in triangular pyramid with the neighboring S atoms, both showing low electronic density near the Fermi level, thus, resulting in nearly null electrical conductivity. This situation seems to be more favorable for the Seebeck effect.

The specific arrangement in bipyramids and tetrahedrons of the antimony atoms in SbS_2_ favors the electron transport in the *x* and *y* direction of the cell. Alloying SbS_2_ with Zn results in a 6.7% decrease in cell volume. Along the *z* direction, the good electron conduction is attributed to the presence of ZnS_4_ and SbS_4_ polyhedra and to the dominance of the p states of sulphur atoms at the top of the valence band. Overall, the electronic thermoelectric properties of this alloy are good, but the lattice thermal conductivity is predicted to be higher than that in pure SbS_2_. As SbS_2_ is alloyed with gallium, the cell volume increases by about 10%. The arrangement around the antimony atoms is weakly affected. A quite specific band structure is exhibited by this structure with both a single valence band and a single conduction band around the Fermi level, well separated from the other valence and conduction bands. These isolated bands are both contributed by Sb-s and S-p orbitals and the SbS_4_ atomic arrangements constitute the corresponding transport path for electrons from the valence band to the conduction band. As it turns out, the power factor is lower for SbS_2_ alloyed with gallium than for SbS_2_ alloyed with zinc. All in all, the Sb-S compounds show intermediate thermoelectric performances that can be compensated for by their environmental friendliness.

## Figures and Tables

**Table 1:** Calculated structural parameters (Å), volume (Å^3^), and energy band gap (eV) of Sb_2_S_3_, SbS_2_, and their alloys.

Structure	a	b	c	Volume	Energy Gap
Sb_2_S_3_	11.803	3.883	11.289	517.4	1.43
Sb_2_S_3_Be_2_	12.790	3.794	11.588	562.3	0.55
SbS_2_	6.564	6.564	8.141	350.8	0.79
ZnSbS_2_	6.919	6.919	6.839	327.4	0.65
GaSbS_2_	6.939	6.939	8.032	386.7	0.55

## Supplementary Materials

The following are available online at https://www.mdpi.com/1996-1944/13/21/4707/s1, Figure S1: Electrical conductivity as a function of chemical potential in different directions at 300 K for Sb2S3. Figure S2: Thermoelectric properties vs. temperature for Sb2S3 with various carrier concentrations (e/cm3 and holes.cm-3). a) Power factor; b) Electrical conductivity. Negative and positive values of carrier concentrations correspond to n-type and p-type doping, respectively. Figure S3: Energy band structure of Sb2S3Be2. Figure S4: Density of states projected on the atoms involved in the Be pyramid: a) Be atom; b,c,d) S atoms. Figure S5: (a) Electrical conductivity; (b) Seebeck coefficient in different directions as a function of chemical potential for Sb2S3Be2 at 300 K. Figure S6: Thermoelectric properties (Seebeck coefficient (a), electrical conductivity (b) and power factor (c)) as a function of the chemical potential at 300 K for Sb2S3Be2. Figure S7: Density of states (DOS) of Sb2S3Be2. Figure S8: Thermoelectric properties of Sb2S3Be2: (a) Thermoelectric power factor; (b) Seebeck coefficient as a function of temperature for various carrier concentrations (cm-3). Figure S9: Seebeck coefficient of Sb2S3Be2 vs. temperature for various carrier concentrations (cm-3) and directions. Figure S10: (a) Electronic band structure of SbS2; (b) Density of states (DOS) of SbS2. Figure S11: (a) Thermoelectric properties of SbS2 and (b) Electrical conductivity along the different directions (x,y,z) as a function of chemical potential at 300 K. Figure S12: Thermoelectric properties of SbS2 vs. temperature for various carrier concentrations (cm-3) (a) Seebeck coefficient and (b) thermoelectric power factor. Figure S13: (a) Band structure of SbS2Zn2 and (b) Density of states (DOS) of SbS2Zn2.The insert in (b) corresponds to the PDOS of Zn atoms. Figure S14. Electronic properties of SbS2Zn2 (a) electrical conductivity for x,y and z directions; (b) thermoelectric properties vs. chemical potential at 300 K. Figure S15: Thermoelectric properties of SbS2Zn2: (a) Thermoelectric power factor and (b) Seebeck coefficient vs. temperature for various carrier concentrations (e/cm3). Figure S16: (a) Band structure of SbS2Ga2 and (b) Density of states (DOS) of SbS2Ga2. Figure S17: Electronic properties of SbS2Ga2 (a) Thermoelectric properties vs. chemical potential at 300 K; (b) Power factor as a function of temperature for various carrier concentrations (cm-3). Figure S18: Electronic properties of SbS2Ga2 vs. chemical potential at 300 K in different directions (a) Electrical conductivity and (b) Seebeck coefficient.

## References

[1] Yang H., Boulet P., Record M.-C. (2020). New insight into the structure-property relationships from chemical bonding analysis: Application to thermoelectric materials. J. Solid State Chem..

[2] Phillips J.C. (1967). A posteriori theory of covalent bonding. Phys. Rev. Lett..

[3] Phillips J.C. (1968). Dielectric definition of electronegativity. Phys. Rev. Lett..

[4] Phillips J.C. (1968). Covalent bond in crystals, II. Partially ionic binding. Phys. Rev..

[5] Phillips J.C., Van Vechten J.A. (1969). Dielectric classification of crystal structures, ionization potentials, and band structures. Phys. Rev. Lett..

[6] Stiles P.J. (1972). Trends in the ionicity in the average valence V materials. Solid State Comm..

[7] Bader R.F.W., Henneker W.H., Cade P.E. (1967). Molecular Charge Distributions and Chemical Binding. J. Chem. Phys..

[8] Bader R.F.W., Preston H.J.T. (1969). The kinetic energy of molecular charge distributions and molecular stability. Int. J. Quant. Chem..

[9] Bader R.F.W., Stephens M.E. (1975). Spatial localization of the electronic pair and number distributions in molecules. J. Am. Chem. Soc..

[10] Bader R.F.W., Essén H. (1984). The characterization of atomic interactions. J. Chem. Phys..

[11] Skinner B.J., Luce F.D., Makovicky E. (1972). Studies of the sulfosalts of copper III. Phases and phase relations in the system Cu-Sb-S. Econ. Geol..

[12] Tesfaye Firdu F., Taskinen P. (2010). Thermodynamics and Phase Equilibria in the (Ni, Cu, Zn)-(As, Sb, Bi)-S Systems at Elevated Temperatures (300–900°C).

[13] Ghosh C., Varma B.P. (1979). Optical properties of amorphous and crystalline Sb_2_S_3_ thin films. Thin Solid Film..

[14] Savadogo O., Mandal K.C. (1993). Low- cost technique for preparing n-Sb_2_S_3_/p-Si heterojunction solar cells. Appl. Phys. Lett..

[15] Yang R.X., Butler K.T., Walsh A. (2015). Assessment of hybrid organic-inorganic antimony sulfides for earth-abundant photovoltaic applications. J. Phys. Chem. Lett..

[16] Kondrotas R., Chen C., Tang J. (2018). Sb_2_S_3_ Solar Cells. Joule.

[17] Wang Q., Chen Z., Wang J., Xu Y., Wei Y., Wei Y., Qiu L., Lu H., Ding Y., Zhu J. (2019). Sb_2_S_3_ solar cells: Functional layer preparation and device performance. Inorg. Chem. Front..

[18] Cao Y., Zhu X., Jiang J., Liu C., Zhou J., Ni J., Zhang J.P. (2020). Rotational design of charge carrier transport layers for optimal antimony trisulfide solar cells and its integration in tandem devices. Sol. Energy Mater. Sol. Cells.

[19] Lei H., Chen J., Tan Z., Fang G. (2019). Review of Recent Progress in Antimony Chalcogenide-Based Solar Cells: Materials and Devices. Solar RLL.

[20] Guo H., Hou W., Liang B., Zhang H. (2017). Fabrication and Photocatalytic Performance of Sb_2_S_3_ Film/ITO Combination. Catal. Lett..

[21] Hosseini M., Pourabadeh A., Fakhri A., Hallajzadeh J., Tahami S. (2018). Synthesis and characterization of Sb_2_S_3_-CeO_2_/chitosan-starch as a heterojunction catalyst for photo-degradation of toxic herbicide compound: Optical, photo-reusable, antibacterial and antifungal performances. Int. J. Biol. Macromol..

[22] Zhou J., Chen J., Tang M., Liu Y., Liu X., Wang H. (2018). Facile synthesis of an urchin-like Sb_2_S_3_ nanostructure with high photocatalytic activity. RSC Adv..

[23] Xu M., Zhao J. (2018). Facile Synthesis of 1D/2D Core-Shell Structured Sb_2_S_3_@MoS_2_ Nanorods with Enhanced Photocatalytic Performance. Electron. Mater. Lett..

[24] Linsen H., Liangxing Z., Deyu B., Xiaoqing J., Junhua L., Xiaosong S. (2020). Hybrid photo-catalyst of Sb_2_S_3_ NRs wrapped with rGO by C–S bonding: Ultra-high photo-catalysis effect under visible light. Appl. Surf. Sci..

[25] Nayak B.B., Acharya H.N. (1984). Electrical and thermoelectric properties of antimony(III) sulfide thin films prepared by the dip-dry method. Thin Solid Film..

[26] Ben Nasr T., Maghraoui-Meherzi H., Kamoun-Turki N. (2016). First-principles study of electronic, thermoelectric and thermal properties of Sb_2_S_3_. J. Alloy. Compd..

[27] Clark A.H. (1970). Supergene metastibnite from Mina Alacrán, Pampa Larga, Copiapo, Chile. Am. Mineral..

[28] Brookins D.G. (1972). Stability of stibnite, metastibnite, and some probable dissolved antimony species at 298.15 degrees K and 1 atmosphere. Econ. Geol..

[29] Olivier-Fourcade J., Maurin M., Philippot E. (1983). Étude cristallochimique de système Li_2_S-Sb_2_S_3_. Revue de Chimie Minérale.

[30] Jain A., Ong S.P., Hautier G., Chen W., Richards W.D., Dacek S., Cholia S., Gunter D., Skinner D., Ceder G. (2013). Commentary: The Materials Project: A materials genome approach to accelerating materials innovation. APL Mater..

[31] Perdew J.P., Burke K., Ernzerhof M. (1996). Generalized Gradient Approximation Made Simple. Phys. Rev. Lett..

[32] Giannozzi P., Baroni S., Bonini N., Calandra M., Car R., Cavazzoni C., Ceresoli D., Chiarotti G.L., Cococcioni M., Dabo I. (2009). QUANTUM ESPRESSO: A modular and open-source software project for quantum simulations of materials. J. Phys. Condens. Matter..

[33] Giannozzi P., Andreussi O., Brumme T., Bunau O., Nardelli M.B., Calandra M., Car R., Cavazzoni C., Ceresoli D., Cococcioni M. (2017). Advanced capabilities for materials modelling with Quantum ESPRESSO. J. Phys. Condens. Matter..

[34] Kyono A., Kimata M. (2004). Structural variations induced by difference of the inert pair effect in the stibnite-bismuthinite solid solution series (Sb,Bi)_2_S_3_. Am. Mineral..

[35] Madsen G.K.H., Singh D.J. (2006). BoltzTraP. A code for calculating band-structure dependent quantities. Comput. Phys. Comm..

[36] Otero-de-la-Roza A., Johnson E.R., Luaña V. (2014). Critic2: A program for real-space analysis of quantum chemical interactions in solids. Comput. Phys. Comm..

[37] Kirzhnits D.A. (1957). Quantum corrections to the Thomas–Fermi equation. Sov. Phys. JETP.

[38] Kirzhnits D.A. (1967). Field Theoretical Methods in Many-Body Systems.

[39] Abramov Y.A. (1997). On the possibility of kinetic energy density evaluation from the experimental electron-density distribution. Acta Crystallogr. Sect. A Found. Crystallogr..

[40] Espinosa E., Alkorta I., Elguero J. (2002). From weak to strong interactions: A comprehensive analysis of the topological and energetic properties of the electron density distribution involving X–H⋯F–Y systems. J. Chem. Phys..

[41] Gervasio G., Bianchi R., Marabello D. (2004). About the topological classification of the metal-metal bond. Chem. Phys. Lett..

[42] Gatti C. (2005). Chemical bonding in crystals: New directions. Z. Kristallogr. Cryst. Mater..

[43] Momma K., Izumi F. (2011). VESTA 3 for three-dimensional visualization of crystal, volumetric and morphology data. J. Appl. Crystallogr..

[44] Cope A.D. (1959). Photoconductive Device. U.S. Patent.

[45] Grigas J., Meshkauska J., Orliukas A. (1976). Dielectric properties of Sb_2_S_3_ at microwave frequencies. Phys. Status Solidi A.

[46] Ablova M.S., Andreev A.A., Dedegkaev T.T. (1976). Switching effect in Sb_2_S_3_. Sov. Phys. Semicond. USSR.

[47] Chockalingam M.J., Rao K.N., Rangarajan N., Suryanarayana C.V. (1970). Studies on sintered photoconductive layers of antimony trisulphide. J. Phys. D App. Phys..

[48] George J., Radhakrishnan M.K. (1980). Electrical conduction in coevaporated antimony trisulphide films. Solid State Commun..

[49] Arun P., Vedeshwar A.G. (1996). Phase modification by instantaneous heat treatment of Sb_2_S_3_ films and their potential for photothermal optical recording. J. Appl. Phys..

[50] Salem A.M., Selim M.S. (2001). Structure and optical properties of chemically deposited Sb_2_S_3_ thin films. J. Phys. D Appl. Phys..

[51] Maghraoui-Meherzi H., Nasr T.B., Kamoun N., Dachraoui M. (2010). Structural, morphology and optical properties of chemically deposited Sb_2_S_3_ thin films. Phys. B Condens. Matter..

[52] Dutková E., Takacs L., Sayagués M.J., Balaz P., Kovac J., Satka A. (2013). Mechanochemical synthesis of Sb_2_S_3_ and Bi_2_S_3_ nanoparticles. Chem. Eng. Sci..

[53] Roy B., Chakraborty B.R., Bhattacharya R., Dutta A.K. (1978). Electrical and magnetic properties of antimony sulphide (Sb_2_S_3_) crystals and the mechanism of carrier transport in it. Solid State Commun..

[54] Rajpure K.Y., Bhosale C.H. (2000). Effect of composition on the structural, optical and electrical properties of sprayed Sb_2_S_3_ thin films prepared from non-aqueous medium. J. Phys. Chem. Solids.

[55] Sun M., Li D., Li W., Chen Y., Chen Z., He Y., Fu X. (2008). New photocatalyst, Sb_2_S_3_, for degradation of methyl orange under visible-light irradiation. J. Phys. Chem. C..

[56] Carey J.J., Allen J.P., Scanlon D.O., Watson G.W. (2014). The electronic structure of the antimony chalcogenide series: Prospects for optoelectronic applications. J. Solid State Chem..

[57] Caracas R., Gonze X. (2005). First-principles study of the electronic properties of A_2_B_3_ minerals, with A= Bi, Sb and B= S, Se. Phys. Chem. Miner..

[58] Nasr T.B., Maghraoui-Meherzi H., Abdallah H.B., Bennaceur R. (2011). Electronic structure and optical properties of Sb_2_S_3_ crystal. Phys. B Condens. Matter.

[59] Onida G., Reining L., Rubio A. (2002). Electronic excitations: Density-functional versus many-body Green’s-function approaches. Rev. Mod. Phys..

[60] Dennis J.H. (1961). Anisotropy of the Seebeck coefficients of bismuth telluride. Adv. Energy Convers..

[61] Cordier G., Schwidetzky C., Schäfer H. (1984). New SbS_2_ strings in the BaSb_2_S_4_ structure. J. Solid State Chem..

[62] Takebe H., Hirakawa T., Ichiki T., Morinaga K. (2003). Thermal stability and structure of Ge-Sb-S glasses. J. Ceram. Soc. Jpn..

[63] Ajalkar B.D., Chigare P.S., Bhosale P.N. Synthesis and study of physico-chemical properties of nanocystalline (Mo:SbS_2_) thin films. Proceedings of the International Conference on Eerging Horizons in Biochemical Sciences and Nanomaterials.

