# Supplementary material for: Thermoelectric Properties of Sb-S System Compounds from DFT Calculations
Source: Materials (Basel). 2020 Oct 22;13(21):4707. doi: 10.3390/ma13214707 (PMC7684470; doi:10.3390/ma13214707)
Supplement: Supplementary file 1 [file materials-13-04707-s001.zip › materials-914200-supplementary.docx]

**Figure S1.** Electrical conductivity as a function of chemical potential in different directions at 300  K for Sb_2_S_3_.

|  |  |
| --- | --- |
| (a) | (b) |

**Figure S2.** Thermoelectric properties vs. temperature for Sb_2_S_3_ with various carrier concentrations (e.cm^-3^ and holes.cm^-3^). **a**) Power factor; **b**) Electrical conductivity. Negative and positive values of carrier concentrations correspond to n-type and p-type doping, respectively.


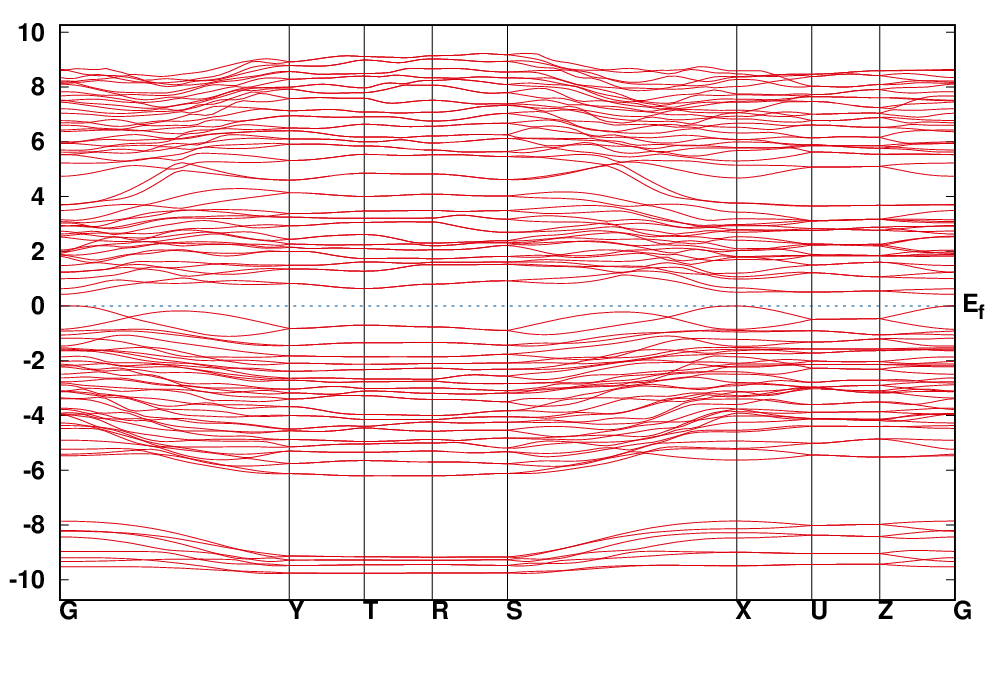


**Figure S3.** Energy band structure of Sb_2_S_3_Be_2_.

|  |  |
| --- | --- |
| (a) | (b) |
|  |  |
| (c) | (d) |

**Figure S4.** Density of states projected on the atoms involved in the Be pyramid : **a**) Be atom; **b**,**c**,**d**) S atoms

|  |  |
| --- | --- |
| **(a)** | **(b)** |

**Figure S5.** (**a**) Electrical conductivity; (**b**) Seebeck coefficient in different directions as a function of chemical potential for Sb_2_S_3_Be_2_ at 300  K.

**Figure S6.** Thermoelectric properties (Seebeck coefficient (**a**), electrical conductivity (**b**) and power factor (**c**)) as a function of the chemical potential at 300 K for Sb_2_S_3_Be_2_.

**Figure S7.** Density of states (DOS) of Sb_2_S_3_Be_2_.

|  |  |
| --- | --- |
| **(a)** | **(b)** |

**Figure S8.** Thermoelectric properties of Sb_2_S_3_Be_2_: (**a**) Thermoelectric power factor; (**b**) Seebeck coefficient as a function of temperature for various carrier concentrations (cm^-3^).

**Figure S9.** Seebeck coefficient of Sb_2_S_3_Be_2_ vs. temperature for various carrier concentrations (cm^-3^) and directions.

| 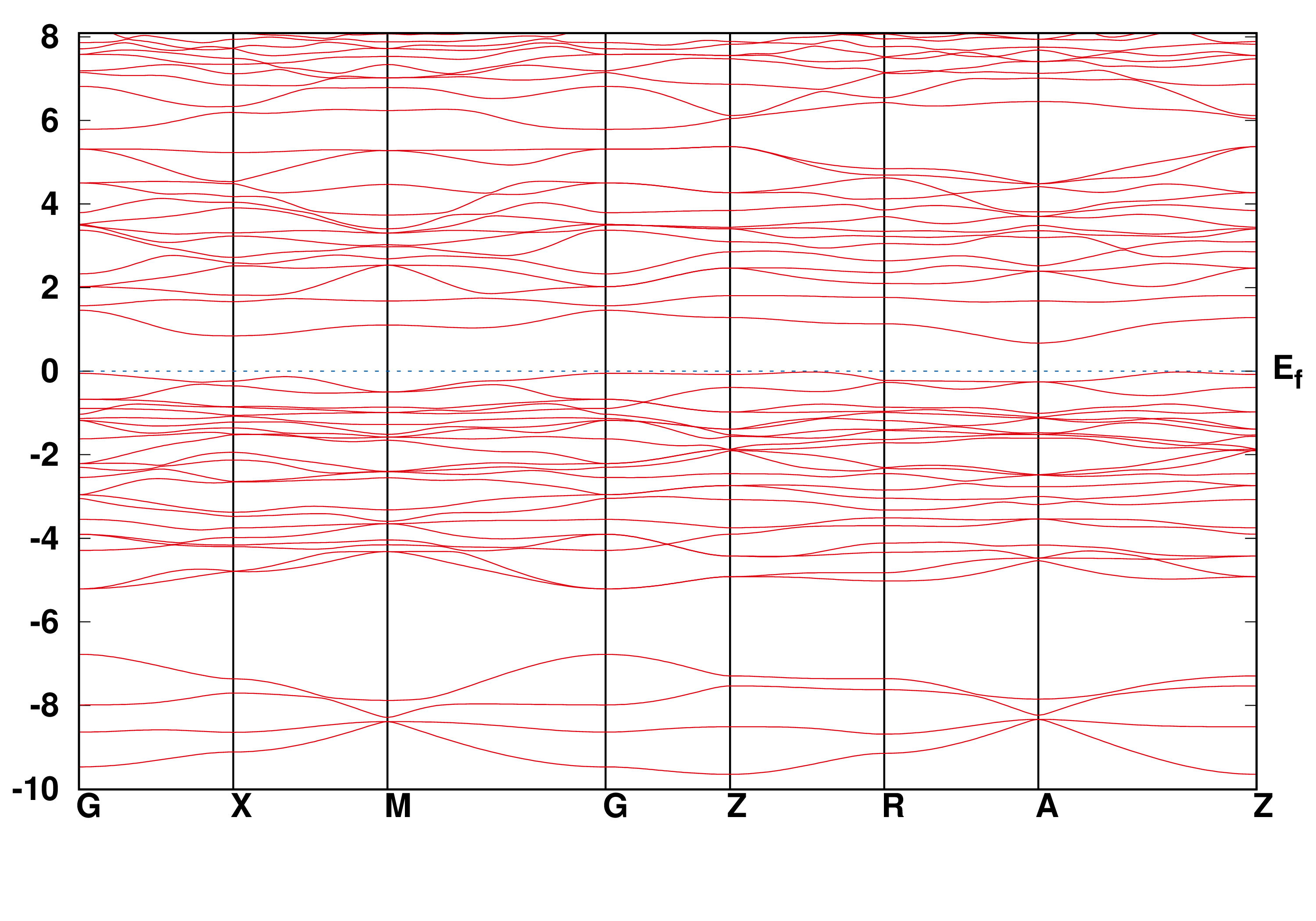 | 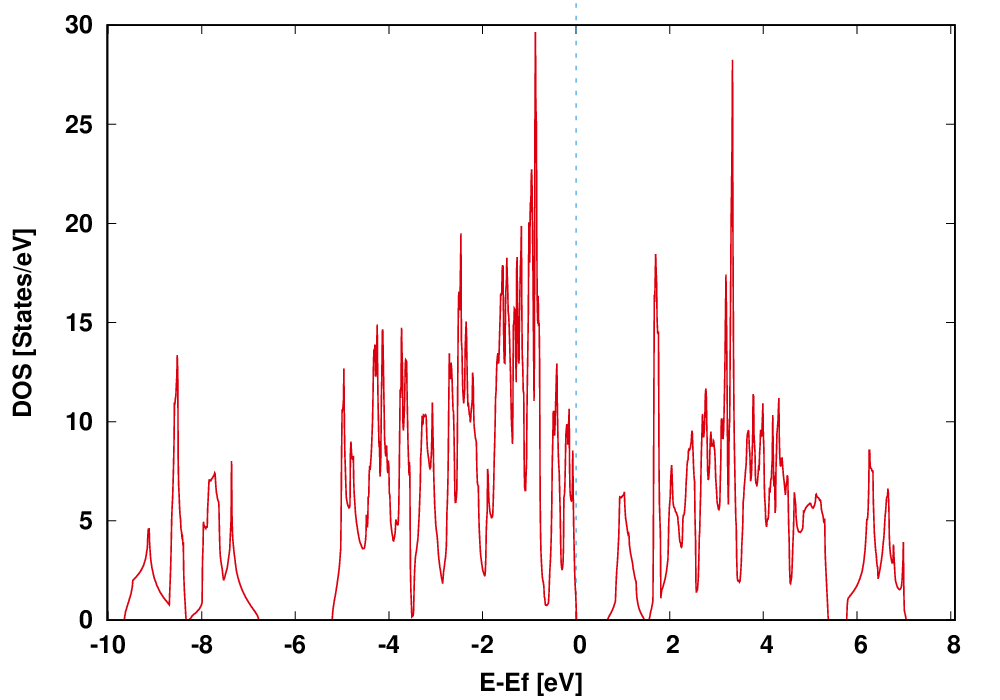 |
| --- | --- |
| (a) | (b) |

**Figure 10.** (**a**) Electronic band structure of SbS_2_; (**b**) Density of states (DOS) of SbS_2_.

|  |  |
| --- | --- |
| (a) | (b) |

**Figure S11.** (**a**) Thermoelectric properties of SbS_2_ and (**b**) Electrical conductivity along the different directions (x,y,z) as a function of chemical potential at 300  K.

|  |  |
| --- | --- |
| (a) | (b) |

**Figure S12.** Thermoelectric properties of SbS2 vs. temperature for various carrier concentrations (/cm3) (**a**) Seebeck coefficient and (**b**) thermoelectric power factor.

| 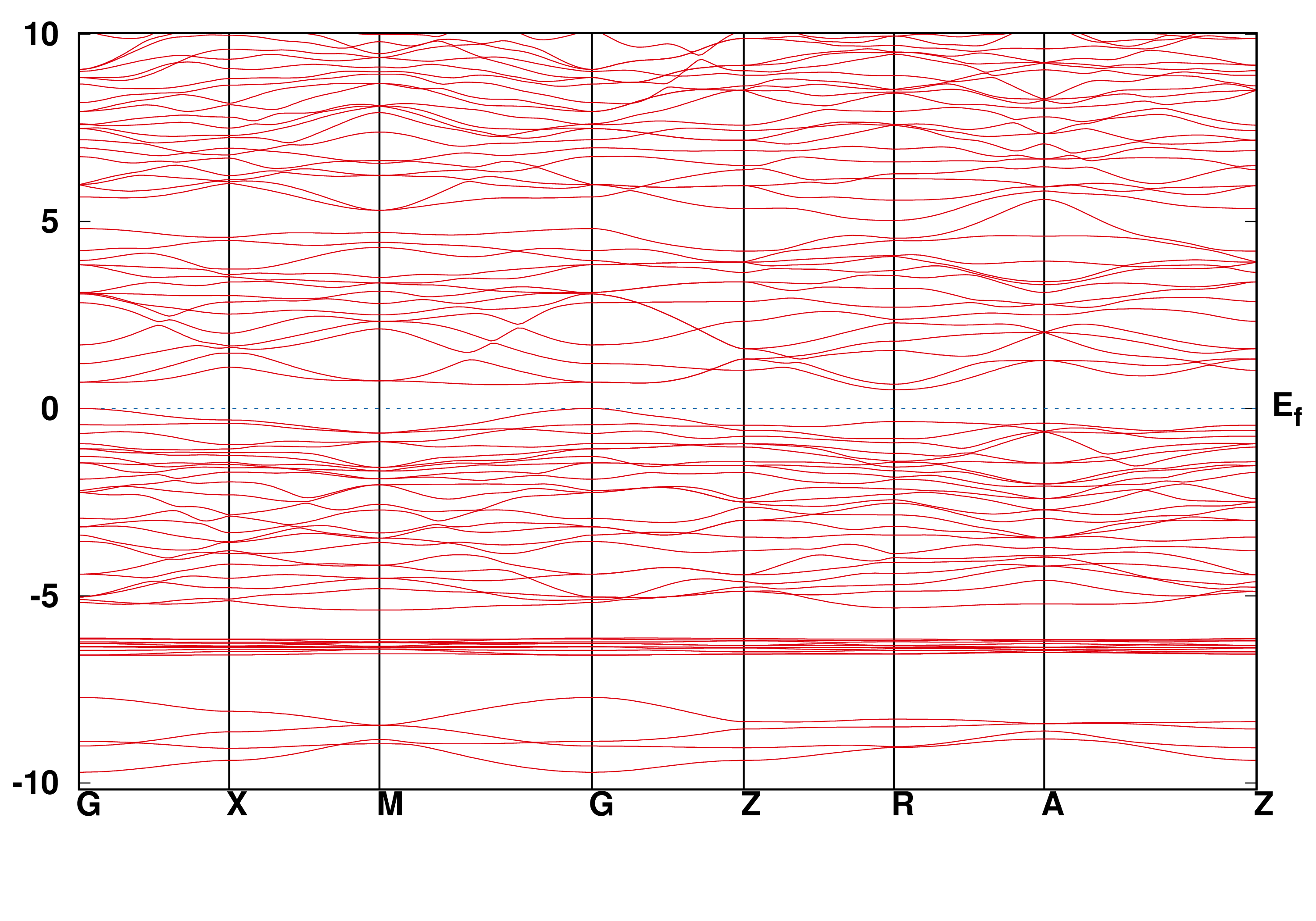 |  |
| --- | --- |
| (a) | (b) |

**Figure S13.** (**a**) Band structure of SbS_2_Zn_2_ and (**b**) Density of states (DOS) of SbS_2_Zn_2_.The insert in (b) corresponds to the PDOS of Zn atoms.

|  |  |
| --- | --- |
| (a) | (b) |

**Figure S14.** Electronic properties of SbS_2_Zn_2_ (**a**) electrical conductivity for x,y and z directions; (**b**) thermoelectric properties vs. chemical potential at 300  K.

|  |  |
| --- | --- |
| (a) | (b) |

**Figure S15.** Thermoelectric properties of SbS_2_Zn_2_: (**a**) Thermoelectric power factor and (**b**) Seebeck coefficient vs. temperature for various carrier concentrations (e/cm^3^).

| 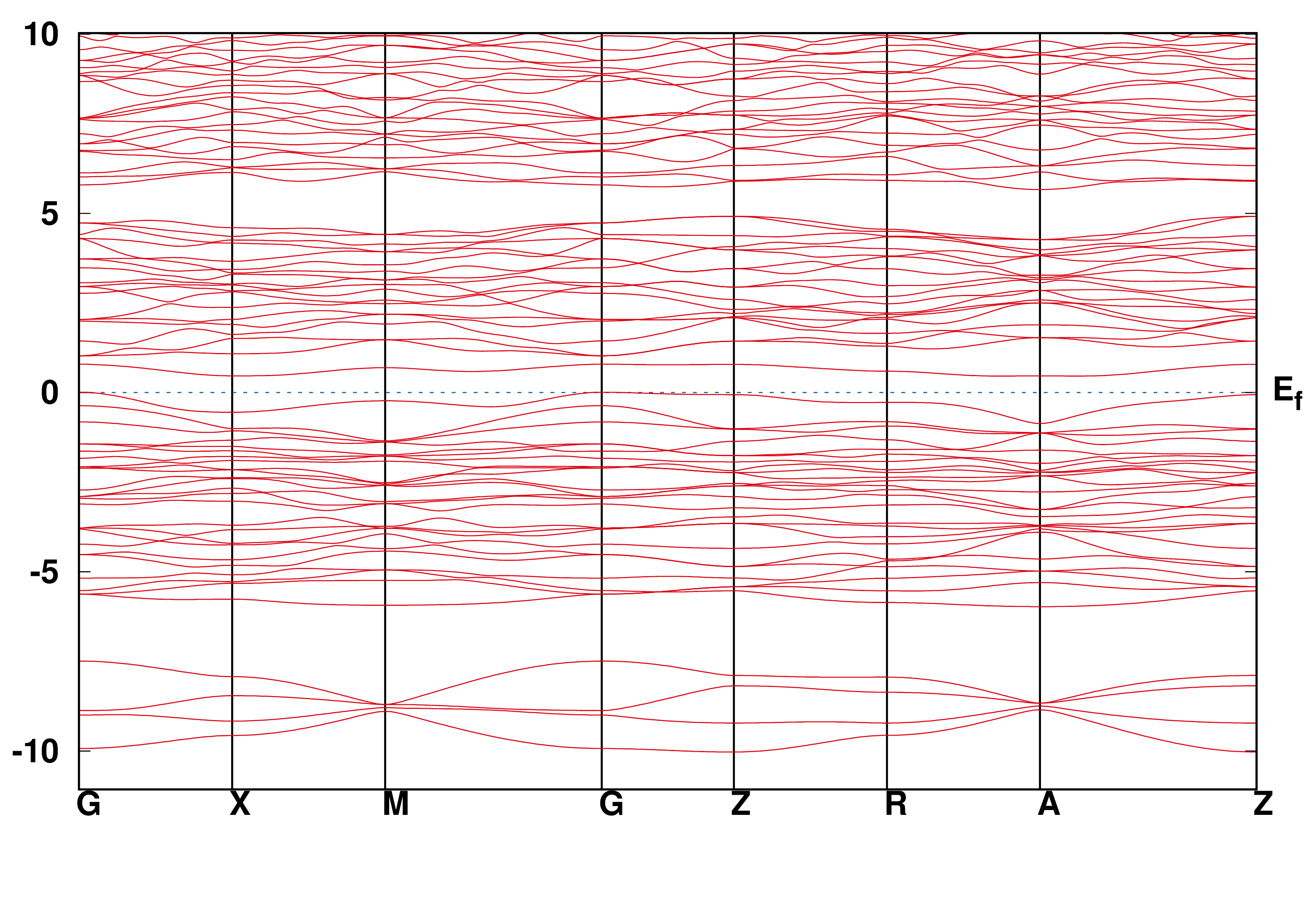 | 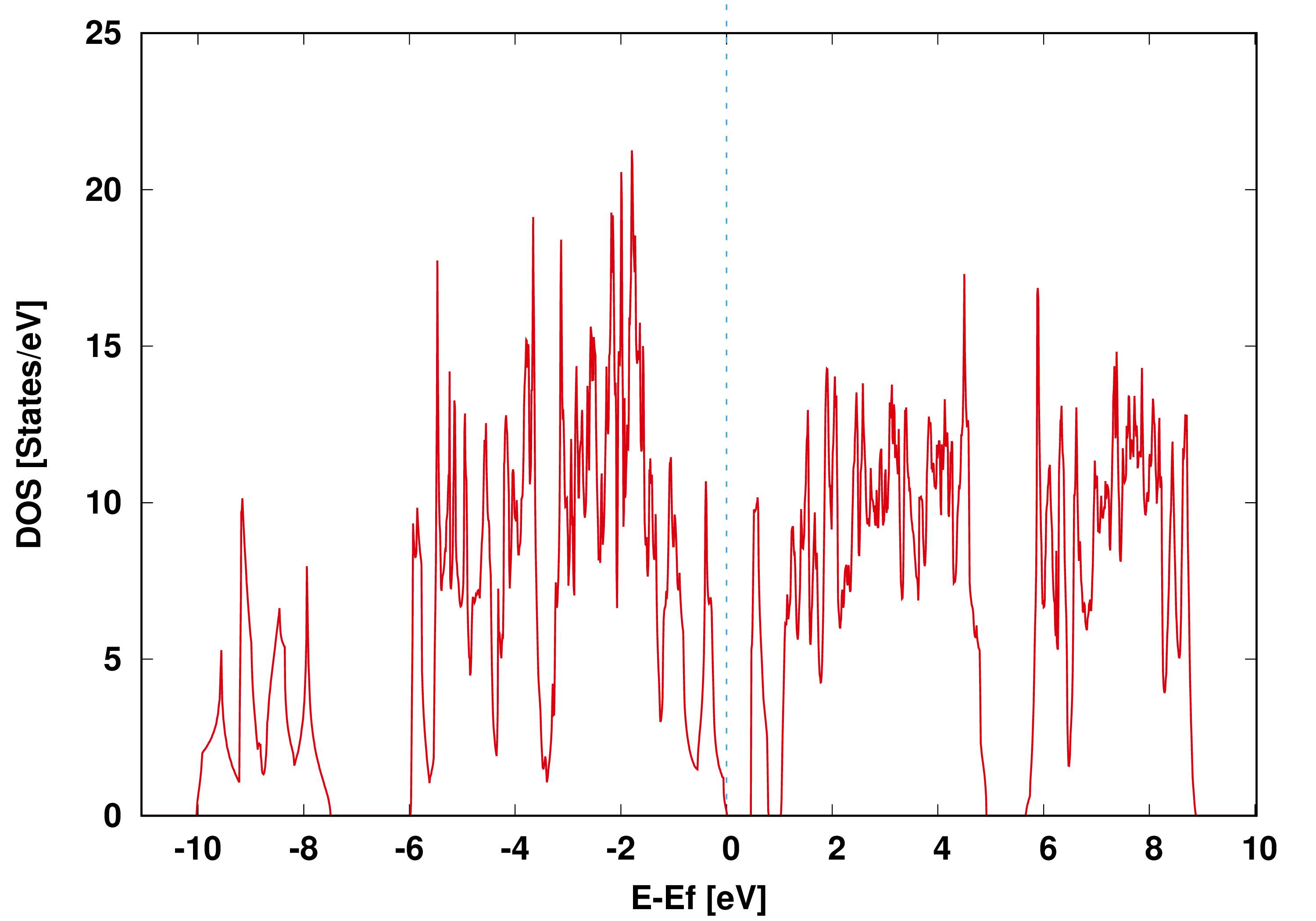 |
| --- | --- |
| (a) | (b) |

**Figure S16.** (**a**) Band structure of SbS_2_Ga_2_ and (**b**) Density of states (DOS) of SbS_2_Ga_2_.

|  |  |
| --- | --- |
| (a) | (b) |

**Figure S17.** Electronic properties of SbS_2_Ga_2_ (**a**) Thermoelectric properties vs. chemical potential at 300  K; (**b**) Power factor as a function of temperature for various carrier concentrations (/cm^3^).

|  |  |
| --- | --- |
| (a) | (b) |

**Figure S18.** Electronic properties of SbS_2_Ga_2_ vs. chemical potential at 300 K in different directions (**a**) Electrical conductivity and (**b**) Seebeck coefficient.
